# Severe Thrombocytopenia Induced by Re‐Exposure to Piperacillin/Tazobactam: A Case Report in Critically Ill Patient With COVID‐19

**DOI:** 10.1002/ccr3.73787

**Published:** 2026-10-30

**Authors:** Abdullah F. Alharthi, Munirah A. Alkathiri, Othman Alomeir, Shmeylan Al Harbi, Ohoud Aljuhani, Haifa A. Alhaidal, Khalid Al Sulaiman

**Affiliations:** ^1^ Department of Clinical Pharmacy, College of Pharmacy Shaqra University Al‐Dawadmi Saudi Arabia; ^2^ Pharmaceutical Care Division King Faisal Specialist Hospital and Research Centre Riyadh Saudi Arabia; ^3^ Pharmaceutical Care Department King Abdulaziz Medical City Riyadh Saudi Arabia; ^4^ College of Pharmacy King Saud Bin Abdulaziz University for Health Sciences Riyadh Saudi Arabia; ^5^ Clinical Trial Management King Abdullah International Medical Research Center Riyadh Saudi Arabia; ^6^ Department of Pharmacy Practice, Faculty of Pharmacy King Abdulaziz University Jeddah Saudi Arabia; ^7^ Pharmaceutical Care Administration King Saud Medical City Riyadh Saudi Arabia; ^8^ Saudi Critical Care Pharmacy Research (SCAPE) Platform Riyadh Saudi Arabia; ^9^ Saudi Society for Multidisciplinary Research Development and Education (SCAPE Society) Riyadh Saudi Arabia

**Keywords:** case report, COVID‐19, critically ill, drug‐induced thrombocytopenia, piperacillin/tazobactam

## Abstract

Drug‐induced thrombocytopenia is a condition where certain medications can significantly reduce the platelets in the blood. This can increase the risk of bleeding and bruising. We reported a case of a 73‐year‐old woman with multiple comorbidities who was admitted to the hospital with COVID‐19. Upon admission, the patient received multiple antibiotics, including Piperacillin/Tazobactam. In the following days, she developed severe thrombocytopenia upon re‐exposure to Piperacillin/Tazobactam. Other causes of thrombocytopenia were systematically excluded. After discontinuing the Piperacillin/Tazobactam and administering intravenous immunoglobulin (IVIG), the thrombocytopenia resolved. A Naranjo Adverse Drug Reaction Probability Scale score was calculated and indicated a probable (i.e., 6 points) adverse drug reaction. This case underscores the importance of considering Piperacillin/Tazobactam as a potential cause of severe thrombocytopenia in critically ill patients, even in the presence of confounding factors such as COVID‐19. To our knowledge, this is a rarely reported presentation in a patient with a recent COVID‐19 infection. Further studies are needed to clarify the relationship between COVID‐19 and drug‐induced thrombocytopenia.

## Key Clinical Message

Drug‐induced immune thrombocytopenia should be considered early in critically ill patients who develop an abrupt and severe decline in platelet count. Prompt recognition and discontinuation of the offending medication are essential. Piperacillin/tazobactam is a recognized but underappreciated cause, particularly following re‐exposure and may result in life‐threatening thrombocytopenia.

## 1 Introduction

Thrombocytopenia is a medical condition characterized by a platelet count of < 150 × 10^9^/L or decreased by 30%–50% from baseline [1]. It is a serious hematological complication frequently observed in hospitalized patients, affecting 13.0% to 44.1% of those admitted to the intensive care unit (ICU) [2]. Additionally, it has been associated with an increased mortality risk, including patients with coexisting coronavirus disease 2019 (COVID‐19) [3, 4]. Thrombocytopenia has multiple etiological factors, including bone marrow suppression, autoimmune diseases, severe infections, and drugs, which necessitate the differential diagnosis in the ICU setting [5].

Among these etiologies, drug‐induced thrombocytopenia (DIT) is a reversible and important cause. It is characterized by the production of drug‐dependent antibodies against platelets, ultimately resulting in platelet destruction by the reticuloendothelial system [6, 7, 8]. It should always be suspected when there is isolated thrombocytopenia without hemolysis or disseminated intravascular coagulation (DIC) [9]. The diagnosis of DIT is quite complex, especially in critically ill patients owing to the multiple contributing factors in this population [10]. Piperacillin/tazobactam, a commonly used broad‐spectrum antibiotic, is a known but infrequent cause of DIT [7, 8, 9, 10, 11]. One systematic review mentioned that of its reported hematologic adverse events, severe thrombocytopenia occurred in up to 37% [12].

The diagnosis is further complicated by COVID‐19 infection, which can independently cause thrombocytopenia through immune‐mediated platelet destruction such as immune thrombocytopenic purpura (ITP) [2, 7, 8, 9]. Here, we present the case of a critically ill patient with a recent COVID‐19 infection who developed severe thrombocytopenia upon re‐exposure to piperacillin/tazobactam, demonstrating the diagnostic and management challenges in this complex clinical scenario.

## 2 Case History

### 2.1 Patient Background and Initial Admission

A 73‐year‐old woman with a past medical history of Type 2 diabetes mellitus, hypertension, atrial fibrillation, heart failure with preserved ejection fraction, pulmonary hypertension, past surgical history of left femur fixation with open reduction and internal fixation complicated by pulmonary embolism, as well as mastectomy 10 years ago for breast cancer. The patient was admitted to the hospital with fever and respiratory symptoms, including cough, shortness of breath, and chest discomfort. One week before admission, the patient was diagnosed with COVID‐19 at another hospital. Her records showed no prior exposure to piperacillin/tazobactam. The chest x‐ray showed bilateral patchy airspace opacity that is highly concerning for infectious processes (Figure 1).

**FIGURE 1:**
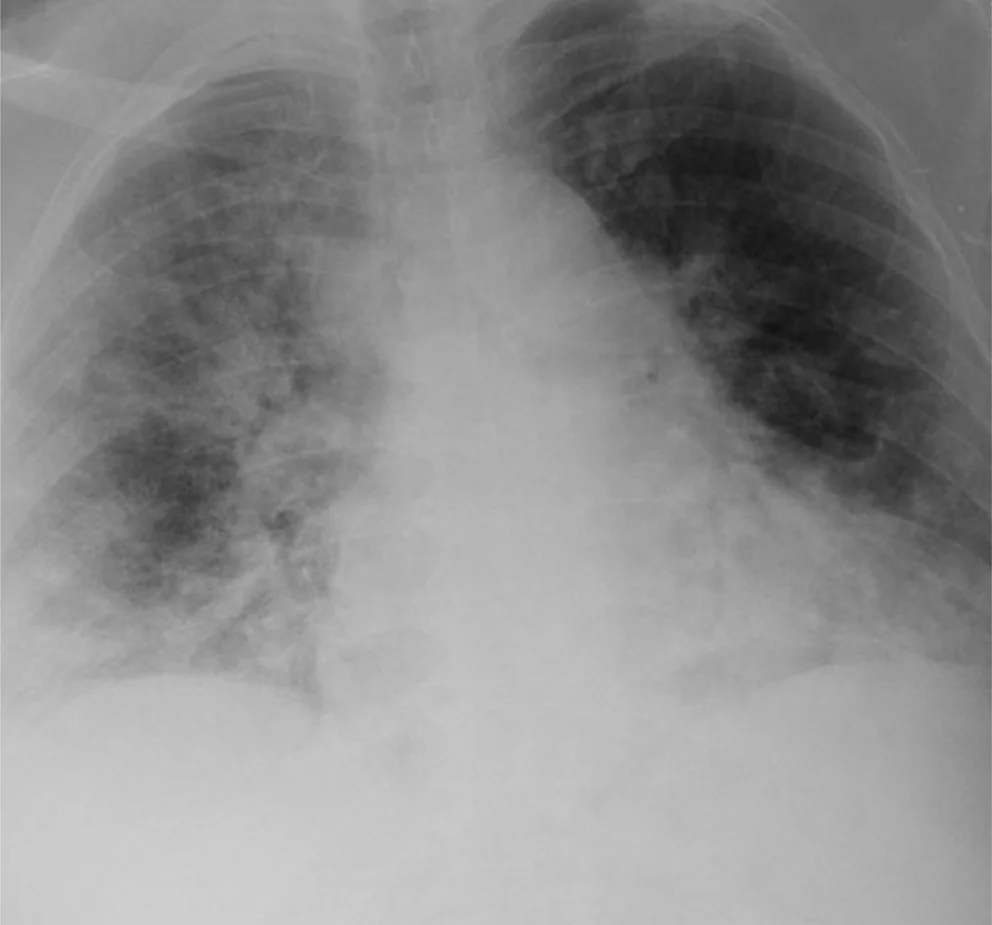
Chest radiograph on hospital admission. Chest radiograph obtained on hospital admission, showing bilateral patchy airspace opacity concerning for an infectious process.

### 2.2 1st ICU Admission

Upon this admission, the patient received a dose of ceftriaxone, then shifted to piperacillin/tazobactam 4.5 g intravenously every 6 h, azithromycin 500 mg orally every 24 h, oseltamivir 75 mg orally every 12 h, and dexamethasone 6 mg intravenously every 24 h. Two days after admission, an electrocardiogram (ECG) revealed a prolonged QTc interval of 528 ms. This led to the discontinuation of azithromycin and the initiation of oral doxycycline 100 mg every 12 h for 6 days. On day 5 of admission, the patient experienced desaturation and tachypnea, with a respiratory rate of 45 breaths/min. Therefore, the critical care response team (CCRT) code was activated, and the patient was transferred to the intensive care unit (ICU). Then, linezolid 600 mg intravenously every 12 h was added to provide coverage for possible methicillin‐resistant *Staphylococcus aureus* (MRSA) infection.

During the third day of ICU admission, the patient remained tachypneic and hypoxic, requiring mechanical ventilation and the administration of a single dose of tocilizumab 350 mg intravenously. On ICU day six, the patient showed improvement and completed a ten‐day course of piperacillin/tazobactam and dexamethasone (stopped after completion of 10 days). During this, the patient's platelet count was around 333 × 10^9^/L without any significant drop. However, the following day, the patient had a spike of fever (39°C) and experienced abdominal discomfort during an examination. Subsequently, a septic workup was obtained because of fever and abdominal discomfort. Although no abdominal imaging was performed, an intra‐abdominal source of infection was considered clinically, and imipenem with caspofungin was added to linezolid empirically. Three days later (day nine), linezolid and caspofungin were discontinued as there was no evidence of Gram‐positive bacterial or fungal infections. On ICU Day 12, the patient developed leukocytosis, with a white blood cell (WBC) count of 21.40 × 10^9^/L, and tracheal aspiration was performed. This led to adding anidulafungin to imipenem due to the suspicion of abdominal perforation. On ICU Day 16, respiratory culture revealed the presence of *Stenotrophomonas maltophilia*, which was susceptible to trimethoprim/sulfamethoxazole. Therefore, anidulafungin was discontinued, and imipenem was substituted with trimethoprim/sulfamethoxazole. On ICU Day 17, the patient was extubated and no longer required inotropes and was subsequently discharged from ICU and transferred to the general ward under the follow‐up of CCRT.

### 2.3 2nd ICU Admission and Thrombocytopenia Event

Four days later, the patient experienced desaturation with an oxygen requirement of 15 L, resulting in readmission to the ICU. On the first day of ICU re‐admission, the patient's WBC count began to increase again, prompting the addition of piperacillin/tazobactam, vancomycin, and caspofungin to trimethoprim/sulfamethoxazole. However, after receiving only two doses of piperacillin/tazobactam the patient was escalated to meropenem. Two days later, a comprehensive septic workup, including chest radiography, computed tomography of the chest, microbiological cultures, and arterial blood gas analysis, returned negative results (Figure 2). Accordingly, meropenem and vancomycin were discontinued because there was no evidence of an active bacterial infection. On Day 7 of ICU re‐admission, patient completed a course of 7 days of caspofungin and 16 days of trimethoprim/sulfamethoxazole therapy. The Patient's condition began to deteriorate again with leukocytosis (WBC count of 14 × 10^9^/L) and fever (38.5°C) on day 13 of ICU re‐admission, suspecting urinary and/or pulmonary infection. As a result, piperacillin/tazobactam, vancomycin, and trimethoprim/sulfamethoxazole were resumed as empirical treatment. However, 8 h later, the patient's platelet counts dramatically dropped from 537 × 10^9^/L to 6 × 10^9^/L (Figure 3). A detailed summary of the patient's clinical course, laboratory findings, and antimicrobial therapy is presented in Table 1.

**FIGURE 2:**
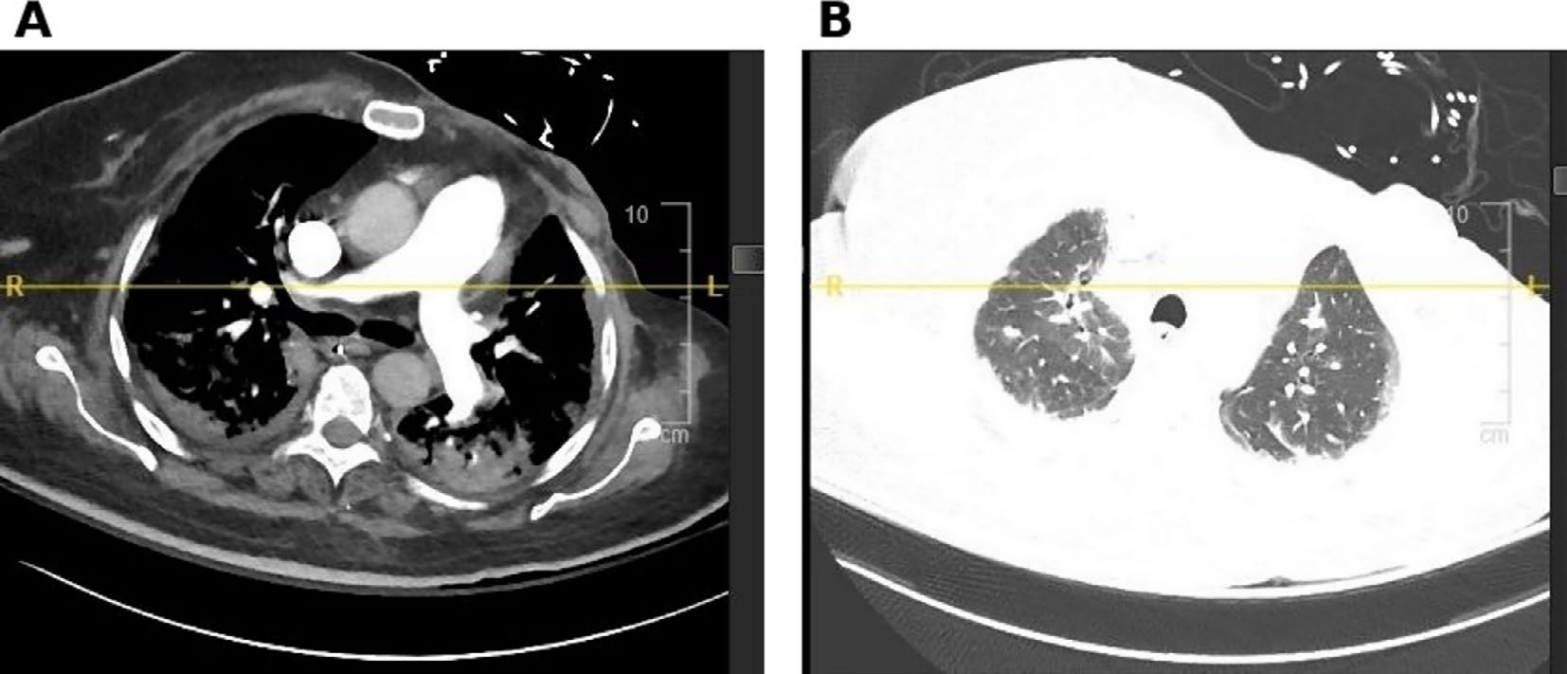
Chest computed tomography during the Second ICU Admission. Chest computed tomography pulmonary angiogram obtained during the second ICU admission as part of the septic workup, showing (A) patent pulmonary vasculature without evidence of embolism, and (B) clear lung parenchyma without evidence of active infection.

**FIGURE 3:**
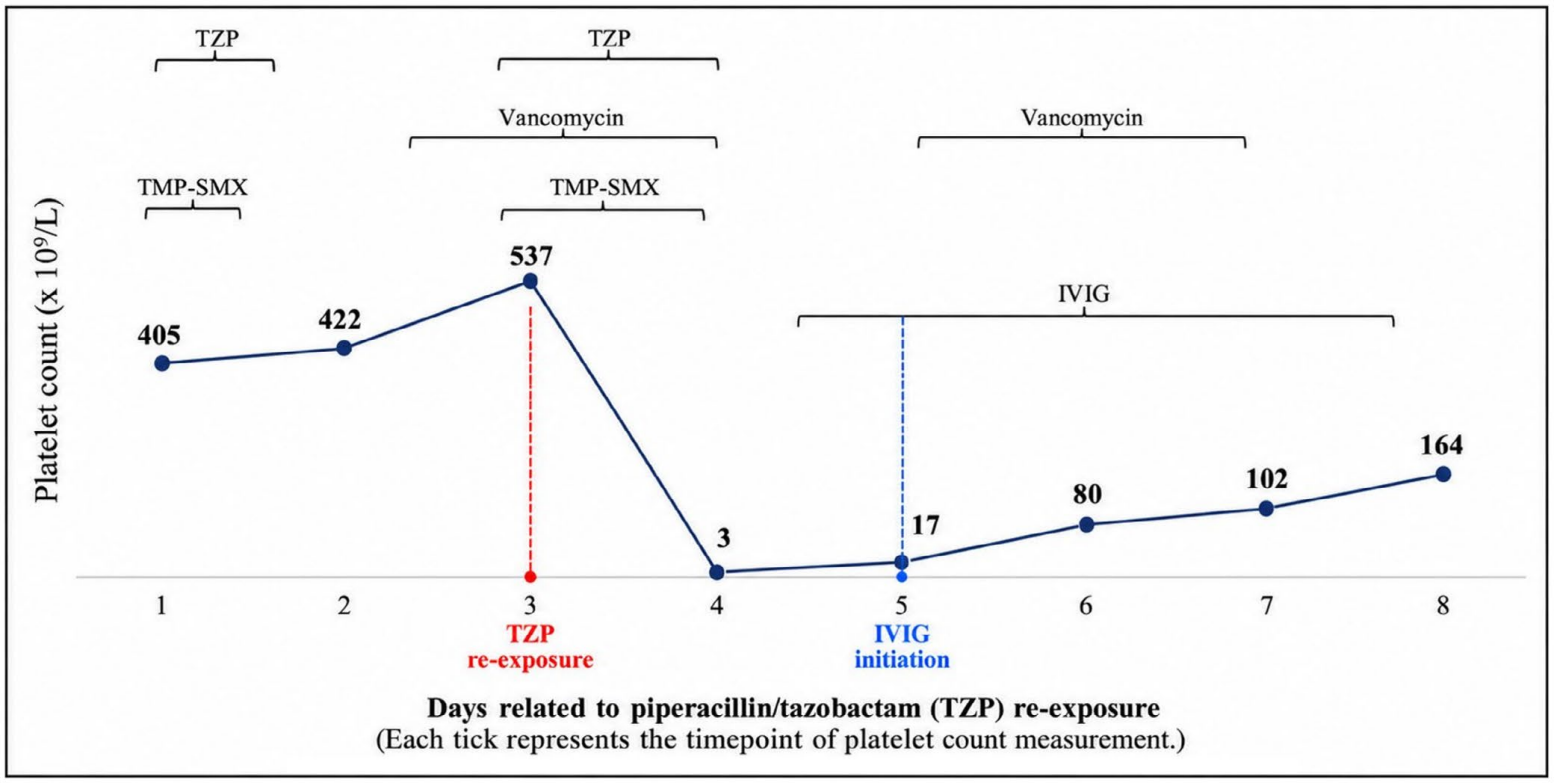
Platelet trend after TZP re‐exposure in the second ICU admission. Each day represents the timepoint of platelet count measurement relative to TZP **re‐exposure** (Day 3). Vertical dashed lines indicate the time of TZP **re‐exposure** and IVIG initiation. IVIG, intravenous immunoglobulin; TMP‐SMX, trimethoprim/sulfamethoxazole; TZP, piperacillin/tazobactam.

**TABLE 1:** Summary of key clinical events and laboratory data.

Day (in ICU)	Setting	Platelet count (×10^9^/L)	Key antibiotics	Major clinical events
1	ICU (1st)	Not reported	TZP, dexamethasone, doxycycline, linezolid	Transferred to ICU (CCRT activated for desaturation/tachypnea); linezolid started for possible MRSA
6	ICU (1st)	~333	TZP, Linezolid, dexamethasone	Completed 10‐day course of TZP + dexamethasone; platelet count stable; fever and abdominal discomfort the following day
16	ICU (1st)	Not reported	TMP‐SMX (substituted for imipenem)	Respiratory culture positive for *S. maltophilia* ; anidulafungin discontinued; imipenem switched to TMP‐SMX
17	ICU (1st)	Not reported	TMP‐SMX	Extubated; inotropes discontinued; discharged from ICU to general ward
Re‐admission Day 1	ICU (2nd)	369	TZP, Vancomycin, Caspofungin, TMP‐SMX	ICU re‐admission; empirical antibiotics
Re‐admission Day 13	ICU (2nd)	537→6	TZP, Vancomycin, TMP‐SMX re‐challenged	Fever: platelet drop after ~8 h
Re‐admission Day 14	ICU (2nd)	3 (nadir)	Meropenem	Offending drugs stopped; IVIG given
Re‐admission Day 15	ICU (2nd)	17	Meropenem	Platelet recovery
Re‐admission Day 35	ICU (2nd)	422	Meropenem Vancomycin re‐challenged	Platelet count normal
Re‐admission Day 36	ICU (2nd)	422	Meropenem Vancomycin re‐challenged	Recurrent *Klebsiella pneumoniae* (ESBL); progressive deterioration with increasing vasopressor requirement; sudden asystolic arrest; patient died. Platelet count remained normal throughout, including during vancomycin re‐challenge, supporting no recurrence of drug‐induced thrombocytopenia

Abbreviations: IVIG, intravenous immunoglobulin; TMP‐SMX, trimethoprim/sulfamethoxazole; TZP, piperacillin/tazobactam.

### 2.4 Differential Diagnosis, Investigations and Treatment

To confirm this extreme drop, a complete blood count (CBC) was repeated, which revealed a platelet count of 3 × 10^9^/L. Normal coagulation studies ruled out DIC, and the absence of schistocytes on a peripheral blood smear, along with normal lactate dehydrogenase (LDH) and haptoglobin levels, excluded thrombotic microangiopathy (TMA) and hemolysis. Piperacillin/tazobactam, vancomycin, and trimethoprim/sulfamethoxazole were all discontinued, and meropenem was initiated. The patient was then given 33 g of intravenous immunoglobulin (IVIG), which resulted in a gradual improvement in platelet count. In addition, platelet transfusions were administered when the platelet count reached a nadir of 3 × 10^9^/L because of the severity of thrombocytopenia. The patient was on *low*‐molecular‐*weight heparin* (LMWH) ICU prophylaxis due to the increased risk of thromboembolic events associated with critical illness. However, after calculating the 4Ts score and confirming the result with the heparin‐induced thrombocytopenia (HIT) test that revealed negative results, heparin‐induced thrombocytopenia was ruled out as the cause of the sudden drop in platelet count. Therefore, the focus shifted to identifying other potential causes, and the hematology team suspected drug‐induced thrombocytopenia, with piperacillin/tazobactam being identified as the most likely offending agent considering the sequence of events and medication administration records. On ICU Day 35, the patient received vancomycin for 2 days without any drop in platelet count. Other medications remained unchanged.

### 2.5 Conclusion and Results (Outcome and Follow‐Up)

After reviewing all medication administration records and considering the sequence of events, piperacillin/tazobactam was identified as the most probable offending agent. Additionally, we have utilized the Naranjo Adverse Drug Reaction Probability Scale for assessing the probability of adverse drug reactions (ADR) [13]. In this case, the patient had a total score of 6, indicating a probable relationship between piperacillin/tazobactam and the development of thrombocytopenia. The scores assigned to individual questions can be seen in Table 2.

**TABLE 2:** Naranjo adverse drug reaction probability scale.

Question	Response	Score
1. Are there previous conclusive reports on this reaction?	Yes	+1
2. Did adverse event appear after the suspected drug was given?	Yes	+2
3. Did the adverse reaction improve when the drug was discontinued, or a specific antagonist was given?	Yes	+1
4. Did the adverse reaction appear when the drug was readministered?	Yes	+2
5. Are there alternative causes that could have caused the reaction?	Yes	−1
6. Did the reaction reappear when a placebo was given?	Not known	0
7. Was the drug detected in any body fluid in toxic concentrations?	Not known	0
8. Was the reaction more severe when the dose was increased or less severe when the dose was decreased?	Not known	0
9. Did the patient have a similar reaction to the same or similar drugs in any previous exposure?	Not known	0
10. Was the adverse event confirmed by any objective evidence?	Yes	+1
Total score	6 (Probable)	

Following recovery of the platelet count, the patient's clinical course remained complicated by her multiple underlying comorbidities, the cumulative effects of prolonged critical illness, and recurrent *Klebsiella pneumoniae* (ESBL) infection. Despite continued antibiotic therapy, she continued to require ongoing intensive care support and did not recover sufficiently for discharge. She subsequently developed a sudden asystolic cardiac arrest while receiving supportive care; resuscitative measures were not pursued in keeping with the clinical context, and she was pronounced dead by the attending physician. Of note, the platelet count had remained stable and within normal limits for an extended period prior to this event, supporting that the fatal outcome was unrelated to piperacillin/tazobactam‐induced thrombocytopenia.

Severe thrombocytopenia induced by medication is a significant complication that can be potentially life‐threatening. This case emphasizes the need for a high index of suspicion for DIT in ICU patients. Piperacillin/tazobactam should be recognized as a potential cause, especially upon re‐exposure. A thorough and systematic approach to rule out other etiologies is essential. Prompt identification and discontinuation of the causative agent are crucial for successful management. Additionally, documenting the occurrence of this adverse reaction can aid in minimizing future exposures and improving morbidity and mortality outcomes.

## 3 Discussion

Drug‐induced thrombocytopenia is a rare but serious adverse effect related to the administration of several medications [11]. It typically develops within a few weeks of initiating a new medication or may present acutely following a single dose if the drug has been taken irregularly in the past [14]. This case illustrates a probable instance of piperacillin/tazobactam‐induced immune thrombocytopenia. The diagnosis is supported by the rapid decline in platelet count immediately after drug re‐exposure, the exclusion of other likely etiologies, and the recovery following its discontinuation. The Naranjo score of 6 further classifies this as a “probable” adverse drug reaction. A systematic review of case reports has shown that piperacillin/tazobactam is associated with a wide range of hematologic adverse effects, with severe thrombocytopenia reported in up to 37% of patients [12]. The exact mechanism by which piperacillin/tazobactam induces thrombocytopenia is not well understood. However, it is hypothesized to induce a reversible change in the conformation of the platelet membrane, resulting in the generation of a neoantigen [12, 15]. This antigen stimulates the synthesis of antibodies against the drug‐platelet membrane complex, leading to the antibody‐mediated destruction of platelets upon re‐exposure to the drug [12, 15]. In our report, factors such as COVID‐19, together with re‐exposure to piperacillin/tazobactam, may have contributed to the development of severe thrombocytopenia.

Trimethoprim/sulfamethoxazole was also considered as a possible contributor because immune‐mediated thrombocytopenia has been reported with this agent [16, 17, 18]. However, thrombocytopenia associated with trimethoprim/sulfamethoxazole typically develops during ongoing therapy and is often accompanied by additional hematologic abnormalities [16, 17, 18]. The temporal relationship observed in our patient more strongly supported piperacillin/tazobactam‐induced thrombocytopenia.

Linezolid was also considered in the differential diagnosis because it is a recognized cause of drug‐induced thrombocytopenia. However, thrombocytopenia associated with linezolid typically occurs after prolonged exposure, often exceeding 10–14 days of therapy [19]. In our patient, linezolid was administered for approximately 9 days and had been discontinued several weeks before the thrombocytopenic episode, making it an unlikely contributor.

Vancomycin was initially thought to be a potential involved agent, but since it was administered within 2 days of the development of thrombocytopenia without any consequences, vancomycin was considered a less likely contributor. In contrast, the patient used trimethoprim/sulfamethoxazole consistently for 16 days, following which she stopped taking it for 2 days before developing thrombocytopenia. The patient subsequently began taking it again on the day thrombocytopenia developed. Despite these findings, it is noteworthy that pancytopenia rather than isolated thrombocytopenia is more frequently linked to trimethoprim/sulfamethoxazole [20]. As well as these additional clinical signs, this drug frequently causes purpura and a variety of oral symptoms, such as petechiae of the oral mucosa or spontaneous oral bleeding [21].

Thrombocytopenia is a commonly observed hematological finding in patients with COVID‐19, particularly in those admitted to ICU [22]. The exact mechanisms underlying COVID‐19‐associated thrombocytopenia, which may involve immune‐mediated platelet destruction or bone marrow suppression, are not fully understood. In this case, although the patient had COVID‐19, the severe thrombocytopenia observed was not solely attributed to the viral infection [22]. The patient's platelet count remained stable during initial treatment with Piperacillin/Tazobactam, indicating that COVID‐19 alone was not the primary cause. However, the subsequent re‐exposure to Piperacillin/Tazobactam coincided with the development of severe thrombocytopenia, suggesting a potential association between the medication and platelet count decline. Taken together, the stable platelet count during the initial piperacillin/tazobactam course, the abrupt decline within hours of re‐exposure, and the recovery following discontinuation and IVIG administration most strongly support piperacillin/tazobactam‐induced immune thrombocytopenia; trimethoprim/sulfamethoxazole and vancomycin were considered less likely contributors given the patient's prior tolerance of both agents. The patient's COVID‐19 diagnosis, made approximately 1 week before admission and several weeks before the thrombocytopenic event, may have contributed to platelet dysregulation, but the strong temporal association with drug re‐exposure favors a drug‐related mechanism [22]. To our knowledge, reports of piperacillin/tazobactam‐induced thrombocytopenia in patients with recent COVID‐19 infection remain very limited, and this case adds concrete detail to that sparse literature. Further studies are needed to clarify the mechanisms of drug‐induced thrombocytopenia and its interaction with COVID‐19.

## Author Contributions

**Abdullah F. Alharthi:** conceptualization, methodology, project administration. **Munirah A. Alkathiri:** data curation, methodology. **Othman Alomeir:** formal analysis, investigation. **Shmeylan Al Harbi:** data curation, formal analysis. **Ohoud Aljuhani:** investigation, methodology, project administration. **Haifa A. Alhaidal:** data curation, investigation. **Khalid Al Sulaiman:** conceptualization, investigation, project administration.

## Funding

The authors have nothing to report.

## Ethics Statement

The study was approved in November 2023 by The Institutional Review Board at King Abdullah International Medical Research Center (KAIMRC) (NRC23R/683/10).

## Consent

Written informed consent from the patient was obtained for publication according to journal guidelines.

## Conflicts of Interest

The authors declare no conflicts of interest.

## Data Availability

The data used and/or analyzed during the current study are available from the corresponding author on reasonable request.
